# Dysthymia increases the risk of temporomandibular disorder: A population-based cohort study (A STROBE-Compliant Article)

**DOI:** 10.1097/MD.0000000000004271

**Published:** 2016-07-22

**Authors:** Shang-Lun Lin, Shang-Liang Wu, Shun-Yao Ko, Ching-Hsiang Lu, Diew-Wei Wang, Ren-Jy Ben, Chi-Ting Horng, Jung-Wu Yang

**Affiliations:** a Department of Psychiatry, Kaohsiung Armed Forces General Hospital, Kaohsiung, Taiwan; b School of Medicine, Griffith University, Gold Coast, Australia; c Graduate Institute of Medical Science, College of Health Science, Chang Jung Christian University, Tainan; d Department of Neurosurgery; e Department of General Surgery; f Department of Medicine; g Department of Ophthalmology, Kaohsiung Armed Forces General Hospital, Kaohsiung; h Graduate Institute of Medical Science, College of Health Science, Chang Jung Christian University; i Department of Oral and Maxillofacial Surgery, Tainan Sin Lau Hospital, the Presbyterian Church in Taiwan; j Graduate Institute of Medical Science, College of Health Science, Chang Jung Christian University, Tainan, Taiwan.

**Keywords:** depression, dysthymia, population-based study, propensity score, temporomandibular disorders

## Abstract

Numerous studies have investigated the relationship between depression and temporomandibular disorders (TMD), but the conclusions remain vague. The aim of this study was to examine the causal effect between depression and TMD.

The reporting of this study conforms to the STROBE statement. In this retrospective cohort study, all samples were recruited from a representative subdataset of 1 million insured persons for the year 2005 Longitudinal Health Insurance Database, who were randomly selected from all beneficiaries enrolled in the National Health Insurance program of Taiwan. We used a propensity score and stratified 926,560 patients into 2 groups (propensity1 = 588,429 and propensity2 = 338,131) and 4 cohorts (propensity1 with depression = 18,038, propensity1 without depression = 570,391, propensity2 with depression = 38,656, propensity2 without depression = 299,475) to detect the development of TMD among the depressive and nondepressive patients between 2004 and 2013.

The positive correlative factors of TMD included female, total number of times seeking medical advice (TTSMA) for anxiety state, TTSMA for generalized anxiety disorder, TTSMA for mandible fracture, and TTSMA for unspecified anomaly of jaw size. The propensity2 group was represented by elder and female-predominant patients who used more psychiatric health resources. Among 3 types of depression, only dysthymia (so-called chronic depression) had a causal impact on TMD in the propensity 2 group. In the propensity 2 group, the hazard ratio of dysthymia for TMD measured by Cox's regression was 1.64 (95% confidence interval 1.28–2.09), after adjusting for demographic factors, psychiatric comorbidities, and maxillofacial confounders. The first-onset mean time of TMD as the consequence of dysthymia was 3.56 years (sd = 2.74, min = 0.08, median = 2.99, max = 9.73).

This study demonstrates that dysthymia increases the risk of TMD in elderly and female-predominant patients who use more psychiatric health resources.

## 1 Introduction

Temporomandibular disorders (TMD) are an enormous public health problem, with a prevalence that ranges from 18% to 35% in the general population.^[1]^ TMD is the second most common musculoskeletal pain, secondary only to chronic lower back pain.^[2]^ In an epidemiological study of Scandinavia and Northern Europe, the lifetime prevalence of TMD reached 93%.^[3]^ A nationwide survey of Dutch adults revealed that 21.5% of the adult population reported TMD, but 85% of these considered no need for treatment, and only 15% of those sought help.^[4]^ Most TMD patients sought treatment due to pain involving the pre-auricular region, jaw, head, and neck.^[5]^ Fernandes et al^[6]^ recently reported that bodily pain complaints might also play an important role in the presence of TMD pain in adolescents. TMD has been considered a multifactorial disease.^[7]^ The common dental comorbidities of TMD include mandible fracture,^[8]^ impaction and received odontectomy,^[8,9]^ and abnormal jaw size with malocclusion.^[10,11]^ Psychological factors might play a major role in TMD.^[12]^ Schiffman et al^ [2]^ recommended a new dual-axis diagnostic criteria for TMD (DC/TMD), which includes 12 common physical diagnoses (Axis I) and psychosocial status evaluation (Axis II).

Depression has become a global disease with an increasing prevalence over time; in 2010, it was the second leading cause of disability worldwide.^[13]^ In a study of adolescents with signs and symptoms of TMD, depression existed in 26.71% of subjects.^[14]^ In another study, assessment of psychological status revealed that 39.8% of patients with TMD experienced moderate to severe depression.^[15]^ Several studies have reported that painful temporomandibular disorders are strongly associated with high levels of depression.^[16–20]^ Furthermore, they are often bi-directional and co-existent.^[16,17]^ However, these studies were unable to prove a causal relationship because of the cross-sectional study design and case-control study with small sample size. These studies did not consider the fact that anxiety disorders and depression share the same serotonin circuitry.^[21]^

A prospective cohort study found that depression, perceived stress, and mood were associated with pain sensitivity and were predictive of 2- to 3e-fold increases in the risk of TMD (*P* < 0.05).^[22]^ However, the study was limited by its small sample size and inclusion of only 1 female. To our knowledge, only 1 population-based cohort study has proved a temporal relationship between depression and TMD.^[7]^ However, the authors were unable to determine which type of depression would induce TMD and failed to adjust for crucial confounders of comorbidities in dental illness and anxiety disorders.

Propensity score, an adequate method for population allocation, can both maximize the differences between groups and minimize the differences within groups. The bias of an observational study can be minimized by propensity score analysis and adjustment for key confounders.^[23,24]^ No current TMD studies have used propensity score for allocating a large population and thereby maximizing the internal and external validity.

The purpose of this study was to investigate the causal effect between depression and TMD. We hypothesized that depression is one of the risk factors of TMD, and patients with depression have more opportunities to be diagnosed with and treated for TMD compared with the general population. This study used propensity scoring and stratified the claims data from The National Health Insurance program of Taiwan into 2 groups (propensity1 and propensity2) and 4 cohorts (propensity1 with depression, propensity1 without depression, propensity2 with depression, propensity2 without depression) to detect the development of TMD among depressive and nondepressive patients.

## 2 Materials and methods

The STROBE guidelines were used to ensure the reporting of this observational study.

### 2.1 Data resources

This study used the claims data of The National Health Insurance program of Taiwan, which has covered >98% of the 23 million people in this population and has contracted with >93% of hospitals and clinics since 1996. The Department of Health National Health Research Institute (NHRI) managed all medical claims data recorded from the contracted health care institutions. All sampled subjects were retrieved from the Longitudinal Health Insurance Database (LHID2005). The LHID2005 includes all of the original medical claims and registration files for 1 million enrollees in the National Health Insurance (NHI) program. The 1 million enrollees in the LHID2005 were randomly selected from all insured persons registered in the 2005 registry of beneficiaries (N = 23.72 million).^[25]^ This dataset consisted of the registry of medical facilities, details of inpatient orders, ambulatory care (including outpatient departments of hospitals or clinics), dental services, and prescriptions linked with anonymized patient identification. Many researchers have proved the high validity of these NHI data,^[26,27]^ and thousands of articles utilizing these data have been published in Science citation index (SCI) journals.^[28,29]^ The LHID is exempt from full review by the institutional review board in Taiwan because all patient identifications were deidentified and released to the public for research purposes. We still obtained an ethical certificate (protocol number SLH919-104-007) from the Ethics Committee of Tainan Sin Lau Hospital, the Presbyterian Church in Taiwan.

### 2.2 Study samples

**Figure 1:**
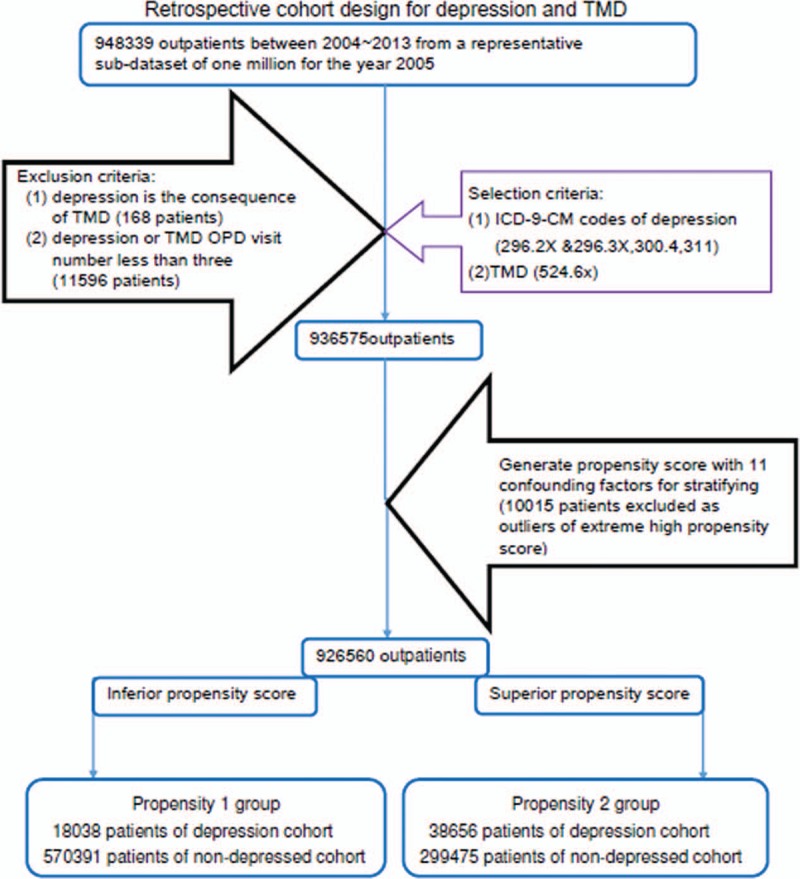
Flowchart of sample selection. Note: OPD = outpatient department.

In selecting samples analyzed in this retrospective cohort study, we first recruited 948,339 outpatients between 2004 and 2013 from a representative subdatasets of 1 million for the year 2005. The samples were selected by using (ICD-9-CM) (International Classification of Diseases, 9th Revision, Clinical Modification) codes for depression (ICD-9-CM code 296.2X, 296.3X, 300.4, and 311) and TMD (ICD-9-CM code 524.60, 524.61, 524.62, 524.63, 524.69). In our study design, the independent variables were 3 common types of depression: major depressive disorder (ICD-9-CM code 296.2X and ICD-9-CM code 296.3X), dysthymia (ICD-9-CM code 300.4), and depressive disorder not elsewhere classified (ICD-9-CM code 311). To avoid a selection bias of depression and TMD diagnoses, we only included those patients who had at least 3 appointments for depression and TMD care during the follow-up period after the index date. This study excluded the following cases: depression as the consequence of TMD (168 patients) and total ambulatory care (including outpatient departments of hospitals or clinics) visits for depression or TMD <3 (11,596 patients). In turn, the remaining cases (936,575 patients) were stratified by the propensity score (10,015 patients excluded) with 11 confounding factors, including age, sex, monthly income, and total number of times of seeking medical advice (TTSMA) for the following illnesses: anxiety state (ICD-9-CM 300.00), panic disorder (ICD-9-CM 300.01), generalized anxiety disorder (ICD-9-CM 300.02), obsessive compulsive disorders (ICD-9-CM 300.03), psychiatric diseases (ICD-9-CM 290-319, except depression) as other psychiatric comorbidities, mandible fracture (ICD-9-CM 802.2, 802.3), impaction (ICD-9-CM 520.6) and received odontectomy (treatment code 92015, 92016, 92063), and abnormal jaw size plus malocclusion (ICD-9-CM 524.0-524.5). Finally, 926,560 outpatients were allocated into 2 groups and 4 cohorts (Fig. 1).

### 2.3 Study starting point and ending point

For patients without depression, the starting point was the first ambulatory care visit (including outpatient departments of hospitals or clinics); for those with depression, the starting point was time of first diagnosis with depression. For patients without TMD, the end point was the last ambulatory care visit; for those with TMD, the end point was the time of first diagnosis with TMD between January 1, 2004, and December 31, 2013.

### 2.4 Statistical analysis

We compared the 11 (no cases of obsessive compulsive disorders [ICD-9-CM 300.03] were coded) confounders of demographic variables and comorbidities between depression and nondepression patients using the propensity score. Cox's regression analysis with hazard ratios (HR) was used to estimate the risk of TMD associated with depression after adjusting for confounders. The onset time of TMD as the consequence of depression was calculated.

All analyses were performed with SPSS statistical software (version 20 for windows; IBM; NY).

## 3 Results

Table 1 compares the continuous variables of confounders between patients with/without depression in 2 propensity groups. In the propensity1 group, patients were younger (28.3 y/o vs 51.7 y/o) and underwent odontectomy more often (0.045 times vs 0.020 times). Those in the propensity2 group sought medical advice more frequently for following disorders: anxiety state (nondepression: 1.26 ± 4.54 outpatient department [OPD] visits vs 0.10 ± 0.64 OPD visits; depression: 5.02 ± 8.54 OPD visits vs 0.72 ± 1.71 OPD visits), panic disorder (nondepression: 0.02 ± 0.39 OPD visits vs 0.00 ± 0.05 OPD visits; depression: 0.28 ± 1.53 OPD visits vs 0.02 ± 0.24 OPD visits), generalized anxiety disorder (nondepression: 0.13 ± 1.13 OPD visits vs 0.01 ± 0.14 OPD visits; depression: 0.85 ± 3.00 OPD visits vs 0.10 ± 0.54 OPD visits), and other psychiatric disease (nondepression: 2.30 ± 10.18 OPD visits vs 0.42 ± 2.37 OPD visits; depression: 8.10 ± 16.83 OPD visits vs 2.02 ± 4.49 OPD visits) except depression.

**Table 1:**
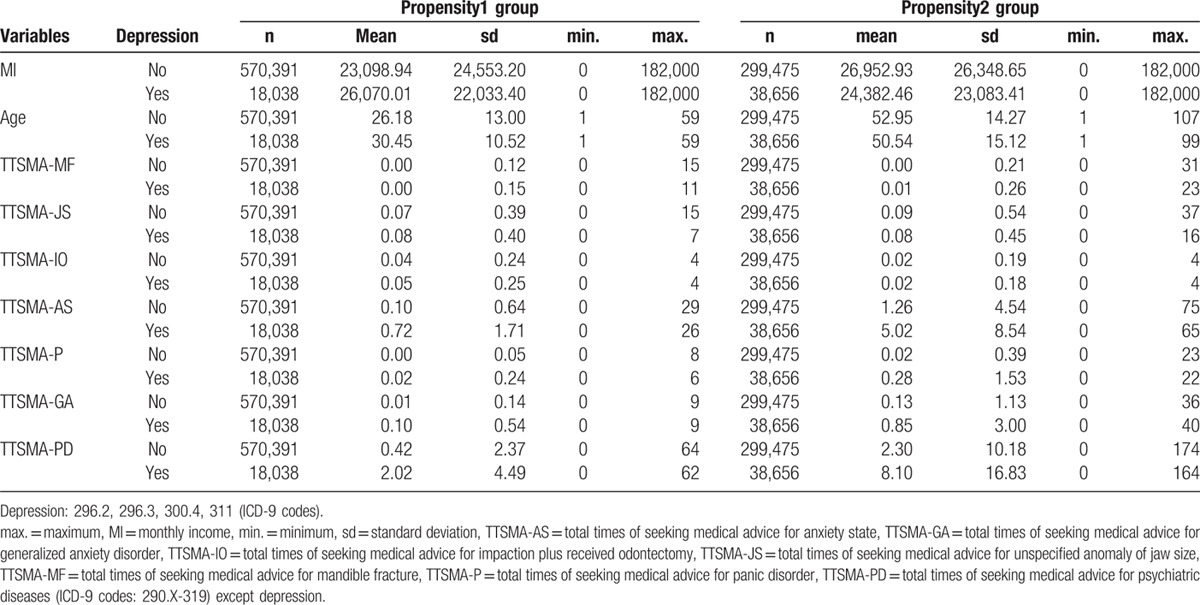
Comparisons in continuous variables of confounders between patients with and without depression for 2004–2013.

Table 2 compares the gender distribution of patients with/without depression in the 2 propensity groups. More women were in the propensity2 group than in the propensity1 group (nondepression: 73.3% vs 36.7%; depression: 72.3% vs 37.4%).

**Table 2:**
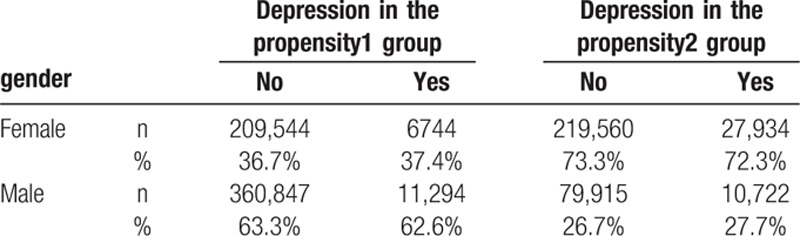
Gender comparisons of confounders between patients with and without depression for 2004–2013.

In Table 3, multivariate Cox's regression analysis shows that the risk of TMD was significantly greater in the dysthymia (ICD-9-CM 300.4) of the propensity2 group (HR 1.64, 95% CI: 1.28, 2.09) under adjusting for 11 confounders. Five confounders demonstrated a significant positive correlation with TMD in both groups: sex (HR 0.42, 95% CI: 0.36,0.50 vs HR 0.59, 95% CI: 0.49,0.71), TTSMA for mandible fracture (TTSMA-MF, HR 1.36, 95% CI: 1.11,1.68 vs HR 1.16, 95% CI: 1.08,1.25), TTSMA for unspecified anomaly of jaw size (TTSMA-JS, HR 1.20, 95% CI: 1.06,1.36 vs HR 1.14, 95% CI: 1.07,1.20), TTSMA for anxiety state (TTSMA-AS, HR 1.16, 95% CI: 1.09,1.23 vs HR 1.03, 95% CI: 1.02,1.04), and TTSMA for generalized anxiety disorder (TTSMA-GA, HR 1.53, 95% CI: 1.30,1.81 vs HR 1.05, 95% CI: 1.02,1.08). Three confounders demonstrated a significant positive correlation with TMD in the propensity1 group: age (HR 1.01, 95% CI: 1.01, 1.02), TTSMA for impaction and received odontectomy (TTSMA-IO, HR 1.74, 95% CI: 1.45, 2.07), and TTSMA for panic disorder (TTSMA-P, HR 1.67, 95% CI: 1.17, 2.41).

**Table 3:**
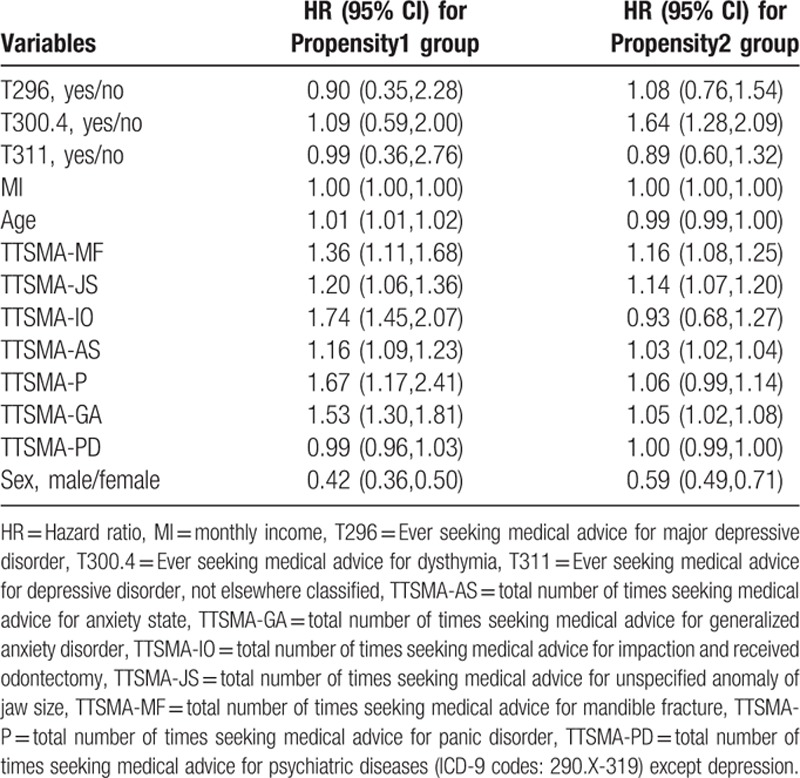
Hazard ratio of temporomandibular disorder in depression adjusted for confounders in Cox's regression analysis.

Table 4 demonstrates that the mean enrolled time was 8.84 ± 2.04 versus 8.55 ± 2.42 years in the propensity1 and propensity2 groups, respectively. In the propensity2 group, the time from dysthymia to onset of TMD was 3.56 ± 2.74 years.

**Table 4:**
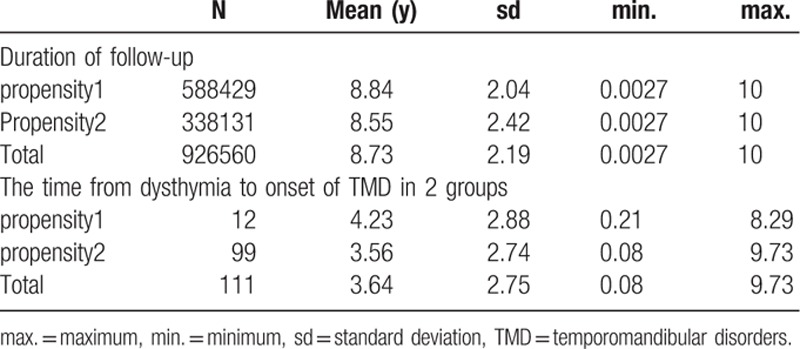
Duration of follow-up and the time from dysthymia to onset of TMD in 2 groups.

## 4 Discussion

There are rare studies investigating the relationship between depression and TMD by using a population-based cohort study design. The aim of our study was to determine whether depression is a risk factor of TMD. To ensure the external validity, the cases were stratified into 2 totally different groups by propensity score, which can absolutely represent 2 age groups and gender groups of 23 million Taiwanese people. To avoid sacrificing internal validity due to large sample size, both psychiatric depressive comorbidities and TMD confounders were adjusted in Cox's regression. This approach not only provides the internal validity but also maximizes the external validity.

Elevated levels of cytokines in the synovial fluid have been associated with inflammation in patients with TMD.^[30]^ Raised cytokine interleukin-8 production capacity is positively associated with severity of depressive disorders.^[31]^ Both TMD and depressive disorders are related to higher levels of cytokines. These findings can explain the association between TMD and depression. Painful temporomandibular disorders have been strongly associated with high levels of depression.^[16,20]^ However, studies showing this are cross-sectional models and thus only present a possible association and not a causal effect relationship. Although Macfarlane et al^[17]^ found in their case-control study that people with PDS (pain dysfunction syndrome) were characterized by high levels of psychological distress, they still could not prove whether depression occurred before the onset of TMD or as a consequence of TMD, as a result of the case-control study design with a small sample size. Slade et al reported that depression was one of the predicted risk factors of first-onset TMD in their “3 years” follow-up prospective cohort study.^[22]^ However, limitations to that study were its small sample size and only female gender. Another prospective cohort study confirmed that psychological distress can predict first-onset of TMD.^[32]^ However, that study collected information of participants based on questionnaires, telephone calls, and/or email, so might not adequately represent clinical diagnoses. Liao et al^[7]^ revealed a temporal relationship between depression and TMD in their population-based cohort study. However, there were still several limitations to their research. First, the definitive criterion of depression was at least 3 visits, but the criterion for TMD was only 1 visit during the follow-up period. Second, the internal validity was diminished by 2 factors: the confounding factors of maxillofacial comorbidities for TMD were not adjusted, and significant difference existed in 6 demographic characteristics between their 2 cohorts. Third, the external validity decreased because their sample size was not enough to represent 23 million Taiwanese people. Fourth, the study did not differentiate which type of depression had the exact causal impact on TMD.

Our study was similar to previous research showing that females have a higher rate of TMD than males,^[7,33]^ and elderly depressive patients also have a higher risk of TMD.^[7]^ These previous studies might explain our findings: the age of propensity1 group was positively correlated with TMD. Concerning the differences between the propensity1 and propensity 2 groups, the propensity2 group had more females, elderly people, depression cases, and a higher use of psychiatric health resources, especially for anxiety disorders. In other words, the propensity2 group represented older female-predominant patients with low serotonin activity.^[21]^ Patients with depression usually have lower serotonin activity than do those with anxiety disorders.^[21]^ The Cox regression analysis of the propensity2 group revealed that dysthymia, so-called chronic depression of lower serotonin activity, had a definite causal effect on older and female patients; the Cox regression analysis of the propensity1 group hinted at a correlative effect of anxiety disorders. The hazard ratio of dysthymia on TMD in propensity2 group was 1.64, which is lower than that of the previous Taiwanese population-based study, at 2.21.^[7]^ This might be because the causal effect of dysthymia was confounded by other subtypes of depression and 11 confounding factors. Our study revealed the existence of a higher risk of TMD problems among the female and the elderly depressive population. The strengths of our study, which has superior validity, are as follows: our study contained 2 completely distinct subgroups, which can represent 23 million Taiwanese people in the domains of gender and age. The study ensures external validity. In addition, our study included a 10-year follow-up period, the definition of depression, and TMD were based on diagnoses by specialists and required at least 3 visits, depression as a consequence of TMD was excluded, and both the psychiatric comorbidities of depression and confounding factors of TMD were adjusted for in Cox regression analysis. All of these factors ensured minimal differences between each group and powerfully increased our internal validity. Therefore, the incidence of TMD in our study is more valid. In addition, we found that only dysthymia (ICD-9-CM 300.4) could induce TMD. This finding hinted at the fact that mood disorder and pain might have similar neurobiological mechanisms and neuro-anatomical substrates, especially chronic mood disorder.^[34]^ Depressive patients had more symptoms of pain and consumed more health resources than nondepressed patients. Therefore, dysthymia (so-called chronic depression) patients with temporomandibular pain had more opportunities to acquire a diagnosis of TMD during the usual process of seeking medical advice. Our findings correspond with the fact that dysthymia patients had the most statistically significant pain symptoms.^[34]^ Successful depression treatment might alleviate the pain of TMD.^[35]^ Our research also determined that the first-onset time of TMD as the consequence of dysthymia (ICD-9-CM 300.4) is 3.56 years (sd = 2.74, min = 0.08, median = 2.99, max = 9.73).

There are still some limitations to our study. First, orthodontic treatment, orthognathic surgery, and partially or completely edentulous patients with denture treatment are not included in The National Health Insurance program, and patients have to pay without assistance from insurance program. Therefore, we could not code the ICD-9-CM from the insurance program, with which our confounding factors of TMD would be more complete. Second, pharmacotherapy for depression and occlusal splint therapy for TMD could not be coded from the NHI program, and these technical problems are still existent; otherwise these treatment codes will help the inclusive criteria more valid. Third, clinical symptoms such as bruxism, which might highly affect TMD, could not code with ICD-9-CM. Fourth, our study design did not allow us to present the causal effects of key confounders. Further studies of different independent variables might help clarify these issues. Finally, we found it difficult to categorize the subtypes of TMD (disc displacement with or without reduction) from the database; therefore, further evaluation of which subtype of TMD is more likely to impact depression is arduous.

## 5 Conclusion

In conclusion, dysthymia increases the risk of TMD in elderly and female-predominant patients who use more psychiatric health resources, and the mean duration between the onsets of the 2 diseases is 3.56 years on average. These results suggest that psychological evaluation should be part of the management of TMD, and these patients should be referred to a psychiatrist as needed. Further cohort studies are needed to assess topics such as whether TMD has a causal effect on depression, how different types of depression influence different subtypes of TMD, and whether maxillofacial comorbidities (e.g., mandible fracture, odontectomy, and anomaly of jaw size) can be causative risk factors for depression.

## Acknowledgments

The authors would like to thank Prof. Shang-Liang Wu for advice about statistical methods, study design, and performance of the calculations.
